# Alzheimer’s Disease and Related Dementias (ADRD) in Rural Communities: Exploring Risks and Pathways to Prevention

**DOI:** 10.1093/geroni/igaf122.020

**Published:** 2025-12-31

**Authors:** Mary (Kate) Clayton-Jones, Mary (Kate) Clayton-Jones

## Abstract

Approximately a quarter of U.S. older adults live in rural communities. Due to limited healthcare access and supportive resources, older adults in rural communities may face an increased risk of disease. However, culturally relevant preventative strategies can help mitigate risks. In this symposium, we will discuss challenges influencing rates of cognitive decline in rural populations and approaches to reduce this disparity. Through an instrument design study, Dr. Cassidy will discuss qualitative findings regarding considerations of usability, inclusivity, and cultural relevance for measuring rural health beliefs toward Alzheimer’s Disease and Related Dementias (ADRD) prevention. Dr. Williams will share ADRD risk factors in a diverse rural population around Lake Okeechobee and correlations observed between cognitive function and factors such as education, hypertension, hearing loss, and social connectedness. Dr. Chanti-Ketterl will discuss the Alzheimer’s Gut Microbiome Project implemented in Indigenous communities in North Carolina, highlighting the success of a mobile clinic research approach to exploring the relationship between cognitive function and the gut microbiome. Dr. Hoffman will report on ADRD early detection and biomarker use in Nebraska and Iowa revealing educational efforts are needed to increase knowledge and use of these important diagnostic tools in rural areas. Lastly, Dr. Ferdows will present on higher ADRD mortality rates linked with restrictive state nurse practitioners and physician assistant policies. Collectively, these studies highlight the need for targeted community and multidisciplinary-centered interventions to reduce dementia risks in rural settings.

